# Potential Impact of Long COVID-19 on Orthodontic Treatment

**DOI:** 10.1055/s-0043-1768467

**Published:** 2023-06-19

**Authors:** Thikriat Al-Jewair, Dimitrios Michelogiannakis, Edmund Khoo, Ryan Prevost

**Affiliations:** 1 Department of Orthodontics, School of Dental Medicine, University at Buffalo, Buffalo, New York, United States; 2 Department of Orthodontics and Dentofacial Orthopedics, Eastman Institute for Oral Health, University of Rochester, New York, United States; 3 Department of Orthodontics and Oral Facial Genetics, Indiana University School of Dentistry, United States; 4 Department of General Dentistry, Eastman Institute for Oral Health, University of Rochester, New York, United States

**Keywords:** long COVID-19, orthodontic tooth movement, orthodontically-induced inflammatory root resorption

## Abstract

Pooled estimates indicate about 226 million individuals are currently experiencing or have experienced persistent symptoms from COVID-19. Long COVID-19 (LC) has been associated with a prolonged inflammatory and stress responses in affected individuals. Due to common pathways, LC could impact the biological mechanisms of orthodontic tooth movement, orthodontically-induced inflammatory root resorption and periodontal tissue response of patients undergoing orthodontic treatment. The authors of the present report discussed potential biological mechanisms through which LC may influence orthodontic treatment highlighting the need for further research in this area.

## Introduction

Coronavirus disease 2019 (COVID-19) is an infectious disease caused by a severe acute respiratory syndrome coronavirus 2 (SARS-CoV-2) identified in 2019. 1 The global number of people infected by COVID-19 surpassed 526 million with a prevalence of 43%. 2 The COVID-19 infection leads to a variety of symptoms such as headache, fatigue, cough, shortness of breath, muscle and joint pain, altered taste and smell, cognitive impairment, and diarrhea; and may even lead to severe pneumonia and death especially in patients with underlying medical conditions. 3 The average recovery time from acute COVID-19 infection ranges between 2 and 3 weeks. 4 This is generally followed by a subacute phase that deals with symptoms that last between 4 and 12 weeks after the onset of illness. 5 6 Symptoms that persist after the acute and subacute infection phases (beyond 12 weeks up to an undefined period) lead to what is currently known as post-COVID-19 syndrome or long COVID-19 (LC). 7

Symptoms of LC include anxiety, fatigue, myalgia, cognitive impairment, sleep disturbances, among others. 3 8 Fatigue and cognitive impairment have a lower incidence in children than adults. 9 Risk factors of LC include being female, older age, high body mass index, history of chronic respiratory disease, and having a severe reaction to COVID-19 during the acute phase. 10 In addition, the presence of more than five symptoms in the first week of acute infection was shown to be significantly associated with the development of LC, independent of the patient's age or sex. 11

Pooled estimates indicate about 226 million individuals are currently experiencing or have experienced persistent symptoms from COVID-19. 2 A recent meta-analysis reported that 80% of people who suffered from COVID-19 experienced one or more long-term symptoms. 12 However, accurate reporting of the epidemiology of LC is restricted by several factors including inconsistencies in the diagnostic criteria, reporting systems, follow-up durations, and demographic characteristics of the examined populations. 7 Nonetheless, the incidence of LC has been reported to range between 30 and 96% depending on the examination times after the acute infection. 7 13 14

The pathophysiological mechanism for LC is poorly understood. However, a potential reason for persistent symptoms could be an overall hyperinflammatory response. 6 In particular, when a patient experiences fatigue due to LC it may persist because of direct viral encephalitis, neurological inflammation, hypoxia, and cerebral vascular disease. 9 From a mental health perspective, animal models and brain analyses of COVID-19 patients postmortem provided evidence that SARS-CoV-2 can also penetrate the blood–brain barrier. 15 This can then result in the brain triggering an immune response that releases interleukins (IL), tumor necrosis factor α (TNF-α), and nitric oxide. 15 In addition, hyperinflammatory state, oxidative stress, cytokine storm, and DNA damage have been hypothesized. 16 The LC can also be a sequalae of harboring the virus in tissue reservoirs across the body, leading to reactivation; there may be cross reactivity of COVID-19 antibodies with host proteins, leading to autoimmune problems; and there could be delayed viral clearance due to immune system exhaustion, an overall mitochondrial dysfunction, and alterations in the microbiome leading to long term health consequences. 6

Various methods have been suggested for the diagnosis/detection of COVID-19 including routine clinical screening for the detection of symptoms and clinical manifestations, and confirmation with laboratory detection methods such as nucleic acid amplification test, real-time reverse transcription-polymerase chain reaction test, rapid antigen detection tests, serological techniques, and computed tomography scan. 17 18 However, accurate and early detection and identification of LC remain problematic, which highlights the need for developing new validated screening questionnaires and interviewing methods to identify persisting symptoms such as fatigue, mood and stress disorders, and other mental health conditions associated with LC. 19

It can be a challenge to manage persistent LC symptoms; this is partly due to the overlap of persistent symptoms that could be a result of mental health problems from the pandemic socially or emotionally leading to fatigue, headache, and other symptoms. 20 Also, it can be a challenge to distinguish between the populations' baseline and their actual LC symptoms, especially for symptoms that present with relatively low prevalence. 21 Thus, it will be very important to consider patients' pre-COVID-19/baseline state.

## LC in Orthodontic Populations

Orthodontic treatment (OT) is commonly performed in children, adolescents, and adults for the improvement of dentofacial esthetics, oral function, and occlusion. 22 23 Since OT and LC are prevalent in the general population (including both growing patients and adults), it is reasonable to assume that a growing number of individuals with LC are currently undergoing or will undergo OT. It has been reported that patients who experience mild or even asymptomatic COVID-19 infection exhibit a prolonged inflammatory and stress response even after 40 days postinfection. 24 In this regard, the authors of the present editorial speculate that the biology and outcomes of orthodontic tooth movement (OTM) might be altered in patients with LC compared with nonpreviously infected or fully recovered individuals. Several studies have assessed the disruption of OT during the COVID-19 pandemic and related lockdown. 25 26 27 These included the disruption of regular patient visits and management of orthodontic emergencies, extended treatment durations, and patient distress and decreased satisfaction with OT during the pandemic. Since it is likely that COVID-19 will continue to have an impact on patients' general and oral health in the foreseeable future, the next section aims to discuss the potential impact of LC on the biological mechanisms of OTM, orthodontically-induced inflammatory root resorption (OIIRR), and periodontal tissue response of patients undergoing OT.

## Potential Impact of LC on OTM

Orthodontically applied forces on teeth create tensile and compressive strains on the surrounding periodontal tissues through a mechanism of mechanotransduction. 28 Specifically, force induced strains at the compression site of the periodontal ligament (PDL) lead to a constriction of the microvasculature (focal necrosis), which manifests histologically as an area of tissue hyalinization. 29 This results in the release of various proinflammatory cytokines including the receptor activator of nuclear factor kappa B ligand (RANKL), TNF-α, IL-1, IL-6, and other prostaglandins and lysosomal enzymes, 28 30 which mediate tissue resorption at the compression site of the PDL. On the other hand, strains at the tension side of the PDL increase blood flow and stimulate alveolar bone apposition by inducing osteoblast progenitor proliferation, reducing RANK signaling, and inhibiting osteoclast activity and formation. 31 In other words, OTM depends on coordinated bone and periodontal tissue remodeling, which is regulated by various biological processes including loading-induced fluid flow, induced hypoxia, and chemical and electrical signaling within the PDL. 31 32 This aseptic inflammatory cascade induced by orthodontic force application is regulated by cytokines, prostaglandins, osteoprotegerin, and other key factors, 31 which enables movement of teeth into the orthodontically-planned positions in patients with malocclusion who undergo OT. Control of the inflammatory process is crucial in patients undergoing OT as unregulated inflammation might lead to side effects including OIIRR, alveolar bone loss, and damage to the dental and paradental tissues. 31 Various factors such as systemic diseases, medications, nicotine, obesity, and stress may influence the inflammatory response to orthodontic force application, 33 34 35 36 37 indicating the need of potential patient counselling, close monitoring, and orthodontic plan/mechanotherapy adaptation in susceptible patient populations.

There has been no substantive research on the impact of LC on OTM. One of the possible routes on how it could affect OTM is via the use of medications to reduce inflammation. Patients with LC are often prescribed long-term anti-inflammatory medications to counter the systemic inflammation. Corticosteroids may also be prescribed in varying durations and strengths. 38 39 The tooth movement pathway is reliant on inflammation, and multiple studies have shown that reducing or blocking inflammation either genetically or through pharmaceutical means can have a profound negative effect on the rate of OTM. 40 41 As such, patients who are on long-term anti-inflammatory drugs have a significant risk of reduced OTM, 42 and this would be an important factor for the orthodontist to take into consideration when formulating the appropriate treatment plan and creating realistic objectives and end-goals for the patient. Furthermore, LC has been associated with a prolonged inflammatory and stress response, 24 and it might affect periodontal tissue remodeling during OT altering the rate of OTM and increasing the risk of periodontal tissue destruction. The above-mentioned hypotheses focused on indirect relationships between LC and OTM. Direct association must not be excluded either. Due to common pathways, LC could impact the biological mechanisms of OTM. Further research is needed to validate these hypotheses.

## Potential Impact of LC on OIIRR and Alveolar Bone Loss

OIIRR and alveolar bone loss are some possible side effects of OT. It has been shown that root resorption can be affected by the amount of orthodontic force, direction of force, and duration of OT. 43 It has also been reported that obesity, respiratory disease, infections, and chronic inflammation can increase the risk of root resorption. 44 45 For these reasons, patients suffering from LC may be at greater risk of OIIRR. At a cellular level, clastic cells such as osteoclasts and cementoclasts have both been implicated in the resorption of the external root surface. It has been demonstrated that there is a possible link between COVID-19 and inflammatory cytokines, especially IL-1, IL-6, and TNF-α which stimulate osteoclast activity, favoring bone resorption through the RANK/RANKL system. It has also been postulated that the COVID-19 virus may also act directly on bone resorptive units. 39 Whether these create a direct risk to OTM and related OIIRR is still not clear; however, the implications still need to be acknowledged.

Through these inflammatory and bone resorptive mechanisms, the risk of bone loss during OTM in patients with LC may potentially be higher. In addition, a dysfunction and alteration of the oral microbiome has been reported in patients with LC. 46 These patients had significantly increased populations of microbiota that induced inflammation, such as members of the genera Prevotella and Veillonella, which are bacterial species that produce lipopolysaccharides. This may also contribute to a greater risk of bone loss and periodontal disease.

## Challenges and Future Research

Undoubtedly, the long-term impact of COVID-19 in the form of LC is still being studied in many fields of medicine and it is pertinent to understand its implications in dentistry and orthodontics. Potential research may include but not limited to retrospective data from orthodontic patients who have had COVID-19 infections and are still suffering from its prolonged state as LC including radiographs or other forms of imaging that may demonstrate impact on OIIRR and bone loss. Similar retrospective research may also be conducted to see if patients with LC who had undergone OT were subjected to longer treatment durations, poorer treatment outcomes, and poor oral hygiene. Furthermore, developing validated screening methods of LC and implementing them in the clinical orthodontic setting would be beneficial for the development of prospective clinical studies that assess the impact of LC on clinical orthodontic outcomes in patients undergoing OT. Such studies will help develop clinical protocols for the orthodontic management of patients with LC to ensure successful orthodontic outcomes while managing the risk of possible side effects.

## Conclusion

The impact of LC on OTM and related parameters remains unclear. The authors of the present review discussed potential biological mechanisms through which LC may influence OTM, OIIRR, and periodontal tissue response to orthodontic force application highlighting the need of further research in this respect.
